# Thyroid and cardiovascular diseases

**DOI:** 10.55730/1300-0144.5927

**Published:** 2024-10-01

**Authors:** Sinem Başak TAN ÖKSÜZ, Mustafa ŞAHİN

**Affiliations:** Division of Endocrinology and Metabolism, Department of Internal Medicine, Faculty of Medicine, Ankara University, Ankara, Turkiye

**Keywords:** Thyroid, hyperthyroidism, hypothyroidism, heart failure, arrhythmias

## Abstract

The thyroid gland is one of the major regulator organs of hemostasis in the human body, controlling the functioning of numerous systems. Thyroid hormones exert a modulating effect on the cardiovascular system in particular, ensuring optimal functioning within the normal range. Triiodothyronine (T3), as an active form of thyroid hormone, is mainly responsible for this effect via both genomic and nongenomic mechanisms. It has been reported that overt thyroid disorders are associated with a number of cardiovascular diseases and cardiac mortality. While hyperthyroidism appears to be related to atrial fibrillation and heart failure, the most pronounced cardiovascular complication of hypothyroidism seems to be atherosclerosis. Achieving euthyroidism is of great importance for restoring cardiovascular function. However, depending on the underlying health conditions, this may not be possible for all patients. Furthermore, there has been a growing focus on the role of subclinical thyroid dysfunctions and their impacts on the cardiovascular system in recent years. The pattern of cardiovascular abnormalities in subclinical thyroid disorders appears to parallel that of overt hypothyroidism, suggesting that even mild alterations in thyroid hormone levels may also have effects on the cardiovascular system. The management of subclinical thyroid disease remains controversial. Current evidence suggests that patient age and underlying cardiovascular diseases are major factors in clinical decision-making.

## 1. Introduction

The thyroid gland is responsible for hemostasis in the human body, regulating the functioning of many different organs and tissues. Any disruption of this critical hemostasis could result in devastating health consequences. More than 10% of the population is affected by thyroid hormone abnormalities, making them some of the most common endocrine disorders worldwide [1]. Although thyroid disorders are generally easy to identify and treat, they may lead to profound clinical implications if they remain undiagnosed. Thyroid hormones are closely related to the cardiovascular system. Some of the most important clinical presentations of thyroid disorders are cardiac manifestations. Moreover, thyroid disorders have been traditionally specified as nonclassical risk factors for cardiovascular diseases. Therefore, thyroid hormone tests have become a routine part of laboratory checklists in cardiology clinics. This review aims to summarize the effects of thyroid disorders on the cardiovascular system and current treatment strategies.

## 2. Actions of thyroid hormones in the cardiovascular system

Thyroid hormones have direct effects on the regulation of cardiovascular hemodynamics and cardiac function through both genomic and nongenomic mechanisms [2]. Myocardial action is mainly due to the active form of the thyroid hormone triiodothyronine (T3), which is converted from tetraiodothyronine (T4) in peripheral tissues. Thyroid hormones have positive inotropic effects derived from the stimulation of beta-thyroid receptors. They increase the expression of alpha-isoforms of myosin heavy chains and upregulate the calcium-dependent ATPase pumps of the endoplasmic reticulum. As a result, the myocardium becomes more rapidly contracted [4]. In addition, thyroid hormones, particularly T3, significantly affect electrophysiological activity by enhancing systolic depolarization and diastolic repolarization. T3 also has a direct effect on the genes that regulate the pacemaker activity of the heart [2]. Traditionally, direct catecholaminergic stimulation was considered to be responsible for the positive chronotropic effect. However, it has been shown that T3 itself induces the upregulation of beta-1-adrenergic receptors in myocytes, which increases cellular sensitivity to the action of catecholamines [4]. At the peripheral level, vascular endothelial and smooth muscle cells express alpha-1-adrenergic receptors. Stimulation of these receptors by T3 reduces peripheral vascular resistance and consequently diastolic blood pressure [3]. Furthermore, both decreased perfusion due to reduced diastolic blood pressure and direct action of T3 activate the renin–angiotensin–aldosterone system, leading to increased sodium retention and circulating volume [4].

## 3. Hyperthyroidism

Hyperthyroidism is diagnosed based on low thyroid-stimulating hormone (TSH) levels with high free T3 and T4, and it affects 0.2%–2.9% of the general population [1]. The three most common etiologies of hyperthyroidism are toxic diffuse goiter, toxic multinodular goiter, and toxic adenoma [5]. Patients with toxic multinodular goiter are more prone to develop cardiovascular complications, which is attributed to toxic multinodular goiter being a disease of the elderly population [6]. A recent metaanalysis of 113,393 hyperthyroid patients demonstrated that overt hyperthyroidism is associated with a 20% increased risk of cardiovascular mortality [7]. Several other studies reported similar results regarding higher hospitalization rates related to cardiovascular diseases and mortality in hyperthyroid patients [7–10]. Once overt hyperthyroidism is detected, appropriate treatment is indicated. However, depending on the general health status of the patient, cardiovascular effects may not fully reverse after normalization of thyroid tests.

### 3.1. Hyperthyroidism and heart failure

As mentioned above, thyroid hormones increase heart rate and myocardial contractility via elevated synthesis of heavy myosin chain proteins as well as increased action of catecholamines [3]. Thus, hyperthyroidism is related to increased cardiac output due to alterations in cardiac rhythm and stroke volume. Data from echocardiographic studies revealed that an excess amount of thyroid hormones enhanced left ventricular systolic function in the short term [11]. Nevertheless, hemodynamic changes due to long-lasting hyperthyroidism paradoxically decrease myocardial contractile reserve, impairing further elevation of ejection fraction and cardiac output in response to exercise [12]. A recent metaanalysis of the echocardiogram findings of 483 patients demonstrated that parameters such as left ventricular ejection fraction, global longitudinal strain, and global circumferential strain are affected when hyperthyroidism persists [13]. The toxic effects of excess thyroid hormones disrupt the myocardial metabolism via alterations in the energy production of the cells and contractile function of myofibrils. Furthermore, they promote relaxation of vascular smooth muscle and decrease vascular resistance, which results in circulatory congestion [3,14]. Although in the early stages of hyperthyroidism left ventricular hypertrophy and increased cardiac output are seen, biventricular dilatation and congestive heart failure dominate the clinical presentation in the later stages.

Manifestations of overt hyperthyroidism include a wide range of cardiovascular signs and symptoms including dyspnea on exertion, tachycardia, and atrial fibrillation. This presentation is referred to as high-output heart failure due to the effects of thyroid hormones on stroke volume and heart rate. Moreover, thyroid hormones activate the renin–angiotensinogen–aldosterone system [15]. As a result, liver congestion and peripheral edema as well as pulmonary congestion due to sodium and fluid retention commonly occur in hyperthyroid patients with heart failure.

A relationship has been documented between thyrotoxicosis and tachycardia-induced cardiomyopathy (tachycardiomyopathy), in which no other underlying cause of heart failure can be identified. Thyrotoxic cardiomyopathy is a specific term that represents the definitive stage of left ventricle dysfunction caused by excessive thyroid hormone. It affects nearly 1% of hyperthyroid patients and is a potentially lethal form of dilated cardiomyopathy that can lead to cardiogenic shock [16].

Correction of thyrotoxicosis has a crucial role in restoring cardiac functions and managing cardiovascular complications. However, selecting a treatment modality is not easy since each one has its own advantages and disadvantages. Radioactive iodine therapy appears to have a deleterious effect on the myocardium and to substantially increase cardiovascular risk [17,18]. Surgical treatment carries the risk of perioperative and intraoperative complications. Thus, medical treatment with antithyroid drugs is often preferred for hyperthyroid patients with heart failure. However medical therapy requires time and carries the risk of recurrence. Symptomatic treatment includes beta-adrenergic blockade to reduce heart rate and diuretics to relieve congestion [19]. Traditionally, cardiomyopathy in hyperthyroid patients was thought to be reversible. However, several studies have noted that it might be persistent depending on the individual characteristics of the patient, such as age, comorbidities, and prior cardiac reserve [20].

### 3.2. Hyperthyroidism and rhythm abnormalities

Patients with hyperthyroidism are at high risk of developing cardiac arrhythmias, the most common of which are sinus tachycardia, atrial fibrillation, and atrial flutter [21]. As mentioned above, thyroid hormones have chronotropic effects on the heart by regulating the transcription of pacemaker-related genes as well as increasing the activation of the beta-adrenergic system in cardiomyocytes. Multiple pathophysiological factors lead to supraventricular arrhythmias including increased left atrial pressure resulting from left ventricular dysfunction, ischemia caused by increased heart rate, and increased atrial ectopic activity [21–23].

The link between hyperthyroidism and atrial fibrillation, which can increase the risk of stroke or heart failure, is well documented. Nearly 5%–25% of patients with hyperthyroidism have atrial fibrillation compared to 1%–3% of the general population. The incidence of atrial fibrillation increases sharply after the age of 60 years [24]. The risk factors for hyperthyroidism-related atrial fibrillation are age, male sex, diabetes mellitus, valvular heart disease, congestive heart failure, and ischemic heart disease [25]. Regarding thyroid function tests, TSH levels are not related to an increased risk of atrial fibrillation but higher T4 levels were shown to be associated with atrial fibrillation [26]. Given the higher incidence of atrial fibrillation in the elderly population, early screening with thyroid function tests is particularly important for this group.

Treatment of thyrotoxicosis is the most critical step in the management of atrial fibrillation. Approximately two-thirds of hyperthyroid patients will spontaneously return to normal sinus rhythm within 3–6 months after achievement of euthyroidism [27]. Therefore, rate control is a priority in symptomatic treatment, while rhythm control is generally reserved for patients with resistant or hemodynamically unstable atrial fibrillation. Although hyperthyroidism itself is associated with a higher risk of coagulation abnormalities [28], evidence regarding whether antithrombotic therapy for hyperthyroid patients with atrial fibrillation has additional benefit is not clear compared to other patients with atrial fibrillation [29]. There may also be a risk of hemorrhage. Thus, anticoagulant therapy in hyperthyroid patients with atrial fibrillation should be guided by the CHA_2_DS_2_-VASc and HAS-BLED scores, as in other patients with atrial fibrillation. A recent metaanalysis demonstrated that oral anticoagulants reduce the risk of ischemic stroke (IS) and systemic embolism (SE) in individuals with thyrotoxic atrial fibrillation when the CHA_2_DS_2_-VASc score is 1 or higher. Direct oral anticoagulants have been shown to offer comparable efficacy to warfarin in the prevention of IS/SE while potentially conferring a lower risk of major bleeding [29].

### 3.3. Hyperthyroidism and pulmonary artery hypertension

Pulmonary artery hypertension is reported to be associated with hyperthyroidism during the disease course. However, the precise mechanism is not yet fully understood and it may be multifactorial. Higher pressure in the left atrium resulting from left ventricular dysfunction is transmitted towards the pulmonary veins, which activates baroreceptors and causes contraction in the arterioles. As the pulmonary artery pressure increases, the right ventricle is forced to eject blood with pulmonary vascularity of greater resistance. If the mean pulmonary artery pressure increases beyond 25 mmHg, this state is called pulmonary artery hypertension [30]. Recent studies have indicated that pulmonary artery hypertension affects 35%–47% of hyperthyroid patients. It is a reversible condition that can be managed effectively when euthyroidism is maintained [31].

### 3.4. Subclinical hyperthyroidism

Subclinical hyperthyroidism is defined as low TSH levels with normal thyroid hormone levels and it affects approximately 0.7%–1.4% of the general population [1]. The cardiovascular outcomes of subclinical hyperthyroidism are similar to those of overt hyperthyroidism with lower severity and frequency. The most important cardiovascular complication of subclinical hyperthyroidism is atrial fibrillation. A recent metaanalysis of 6732 patients with subclinical hyperthyroidism showed an almost twofold increased risk of atrial fibrillation in patients with subclinical hyperthyroidism compared to euthyroid controls [32]. Nevertheless, the current evidence on the risk of stroke in subclinical hyperthyroidism is inadequate [7,33]. Regarding the association between subclinical hyperthyroidism and heart failure, metaanalyses have reported an increased risk of heart failure and cardiovascular mortality, particularly as TSH levels fall below 0.1 mIU/L [34,35]. In light of these findings, current guidelines recommend treatment of subclinical hyperthyroidism when the TSH level is <0.1 mIU/L in patients over 65 years of age and/or with cardiovascular comorbidities [36,37].

## 4. Hypothyroidism

Hypothyroidism is defined as low levels of free T3 and free T4, resulting in elevated levels of TSH. The prevalence of hypothyroidism is reported to be 0.2%–10% in the general population [1]. Cardiovascular complications observed in hypothyroid patients are mainly associated with an increased risk of atherosclerosis and functional cardiovascular abnormalities. A comprehensive metaanalysis of 1,898,314 individuals revealed that patients with hypothyroidism carry higher risks of ischemic heart diseases (13%), myocardial infarction (15%), arrhythmias (96%), and overall mortality (25%) compared to euthyroid patients [38].

### 4.1. Hypothyroidism and atherosclerosis

Thyroid hormone deficiency is associated with an increase in the incidence and severity of atherosclerosis through a variety of mechanisms, including hyperlipidemia, endothelial dysfunction, alterations in blood pressure, coagulation abnormalities, and insulin resistance.

Nearly 90% of hypothyroid patients also suffer from hyperlipidemia [38]. Various studies have reported that in hypothyroidism, levels of total cholesterol, low-density lipoprotein cholesterol (LDL-C), and triglyceride (TG) increase while levels of high-density lipoprotein cholesterol (HDL-C) decrease [40–42]. Hypothyroidism induces hyperlipidemia by increasing total cholesterol synthesis, decreasing the expression of hepatic LDL-C receptors, and inhibiting cholesterol-alpha monooxygenase, the function of which is the clearance of LDL-C [43]. Regarding the high TG levels, hypothyroidism reduces the activity of lipoprotein lipase, which is responsible for degrading TG in circulating chylomicrons [12].

Hypothyroid patients are also at risk of hypertension, which is one of the major risk factors of atherosclerosis. Hypothyroidism impairs vascular smooth muscle relaxation by inhibiting the action of nitric oxide (NO) and causes endothelial dysfunction, which in turn leads to increased arterial stiffness [43]. As peripheral vascular resistance increases, diastolic blood pressure increases and pulse pressure decreases in hypothyroidism.

As the serum TSH level increases, the blood glucose level and insulin resistance level also increase. In a recent metaanalysis, it was shown that hypothyroidism increases the risk of type 2 diabetes mellitus by 26% [44]. In peripheral tissues, the translocation of glucose transporter type 4 (GLUT4) is impaired, resulting in decreased uptake of glucose into cells [45]. In addition, phosphoenolpyruvate carboxin (PEPCK) and glucose-6-phosphate expression are elevated in hypothyroidism, resulting in increased gluconeogenesis in the liver [39].

Hypothyroid patients are susceptible to thromboembolic events because coagulation abnormalities such as increased levels of factor VII and fibrinogen, decreased activation of antithrombin, and fibrinolysis reduction are associated with thyroid hormone deficiency [43]. Moreover, elevated levels of lipoprotein (a) [LP(a)], fructosamine, homocysteine, and C-reactive protein (CRP) have also been reported to further contribute to atherosclerosis in hypothyroid patients [46,47].

### 4.2. Hypothyroidism and rhythm abnormalities

As outlined above, thyroid hormones have significant effects on the pacemaker system of the heart. When thyroid hormone levels are low, a decrease in heart rate is observed. The characteristic findings of electrocardiography include sinus bradycardia, low voltage, and prolonged QT interval in hypothyroid patients [48]. The latter makes hypothyroid patients susceptible to developing atrioventricular blocks or ventricular arrhythmias, such as torsades de pointes.

### 4.3. Hypothyroidism and heart failure

Hypothyroidism can also affect cardiac contractility and impair relaxation of the myocardium. In the early stages, left ventricular diastolic dysfunction is more prominent and systolic function is preserved or minimally impaired. In the later stages, however, both diastolic and systolic functions are disrupted [3]. Consequently, a state of low cardiac output with decreased stroke volume and heart rate may occur. However, rather than the development of new heart failure, the degree of cardiac dysfunction has more to do with the aggravation and worsening of underlying disease [49]. A cohort study of 52,856 participants over 25 years of age found that, compared to the euthyroid group, individuals with hypothyroidism had a significantly increased risk of hospitalization for exacerbation of underlying heart failure (hazard ratio: 1.86) [50]. Additionally, low T3 syndrome, defined as low T3 levels with normal TSH and T4, affects 20%–30% of heart failure patients. It is reported to be associated with poor prognosis and higher risk of all-cause mortality in patients with heart failure [51,52]. Furthermore, severe hypothyroidism is associated with protein-rich pericardial effusion, which may occasionally result in cardiac tamponade. Although the underlying mechanism is not fully understood, increased vascular permeability and decreased lymphatic outflow from the pericardial space have been proposed [11].

### 4.4. Treatment of hypothyroidism

Treatment of overt hypothyroidism with levothyroxine has been shown to ameliorate hyperlipidemia, hypertension, diastolic dysfunction, and bradycardia, thereby delaying the atherosclerotic process [3]. Cardiac contractility and stroke volume may also improve after levothyroxine treatment [48]. Optimal treatment and regular monitoring of TSH is crucial in order to maintain biochemical euthyroidism. A study of a cohort of 216,894 patients in Denmark showed that not only undertreated hypothyroid patients but also overtreated hypothyroid patients are at risk of cardiovascular disease [53]. For patients younger than 50 years of age without heart disease, a dose of 1.6 μg/kg of levothyroxine once daily can be given. However, in older patients with cardiovascular risk factors, it is recommended to start with lower doses and slowly increase to the target dose to avoid precipitating myocardial ischemia or arrhythmias [36,37].

Initial levothyroxine treatment should be begun with daily doses of 0.25 to 0.5 μg/kg in elderly patients. Once the cardiovascular tolerance of the starting dose has been assessed, most experts recommend increasing the daily dose by 12.5 to 25 μg every 4 to 6 weeks until adequate replacement is confirmed by repeat TSH measurement.

### 4.5. Subclinical hypothyroidism

Subclinical hypothyroidism is described as high TSH levels with normal thyroid hormone levels. The severity of subclinical hypothyroidism can be differentiated according to TSH levels, with TSH of >10 mIU/L being considered a severe form whereas TSH of 4–10 mIU/L is defined as a milder form. The prevalence of subclinical hypothyroidism is estimated to be approximately 5%–10% in the general population with a female predominance. While nearly 60% of patients with subclinical hypothyroidism can become euthyroid within 5 years, 1%–5% of patients may progress to overt hypothyroidism annually [54].

The pattern of cardiovascular abnormalities in subclinical hypothyroidism is parallel to that of overt hypothyroidism, suggesting that a milder deficiency of thyroid hormones may also have effects on the cardiovascular system. Cardiovascular changes of endothelial dysfunction, arterial stiffness, diastolic blood pressure, and hyperlipidemia may also occur in patients with subclinical hypothyroidism. As with overt hypothyroidism, subclinical hypothyroidism could result in both diastolic and systolic dysfunction [55]. A metaanalysis of 14 studies concluded that subclinical hypothyroidism increases the risk of cardiac death and/or hospitalization and all-cause mortality in patients with heart failure [56]. Numerous studies have reported an association between subclinical hypothyroidism and an increased risk of ischemic heart diseases and cardiovascular mortality [4,7,38,57]. In a recent metaanalysis of 35 studies including a total of 555,530 individuals, subclinical hypothyroidism was shown to be related to a higher risk of cardiovascular disease (33%) and all-cause mortality (20%). Nevertheless, such relationships were not found for participants older than 65 years [58]. Several of those studies also noted that the increased risk of cardiovascular events appears to be particularly evident when TSH levels are greater than 10 mIU/L and, interestingly, in younger individuals [4,48]. Whether subclinical hypothyroidism may cause accelerated cardiovascular damage in younger individuals is an important question waiting to be addressed.

Treatment of subclinical hypothyroidism when the TSH level is over 10 mIU/L is recommended by the current guidelines [36,37]. However, the treatment of milder forms is still controversial. Randomized clinical trials assessing the impact of levothyroxine (LT4) replacement therapy on cardiovascular outcomes are lacking. Nevertheless, several observational studies have suggested that LT4 therapy improves cardiovascular function and reduces the risk of cardiac mortality, particularly in younger adults [59–61]. Furthermore, in patients with subclinical hypothyroidism, LT4 therapy can improve certain parameters of diastolic function during the observation period [62]. A recent metaanalysis showed that the ratios of E-velocity to A-velocity and global longitudinal strain greatly improved after LT4 therapy [63]. Thus, it is suggested that the decision to apply thyroid replacement therapy should be individualized in young patients with cardiovascular risk factors or diastolic dysfunction. There is a risk of iatrogenic hyperthyroidism, which may lead to cardiovascular complications such as atrial fibrillation and precipitation of heart failure in elderly people. Therefore, thyroid replacement therapy is not recommended until the TSH level rises above 10 mIU/L. When therapy is administered, monitoring TSH levels every 6 to 12 months is recommended [4]. Lower doses of LT4 are usually required to normalize TSH levels compared to overt hypothyroidism.

## 5. Conclusion

Thyroid hormones appear to have a major impact on the cardiovascular system through a variety of pathophysiological mechanisms. Even subclinical forms of thyroid disease can lead to clinical cardiovascular manifestations. Although it is clear that overt hypo- and hyperthyroidism should be treated promptly upon diagnosis, the management of subclinical thyroid disease is still a matter of debate. Based on current knowledge, the age of the patient appears to be an important determinant in clinical decision-making. Younger patients with subclinical hypothyroidism and older patients with subclinical hyperthyroidism seem to derive greater benefit from treatment to reduce cardiovascular complications.
